# Does school attendance reduce the risk of youth homelessness in Tanzania?

**DOI:** 10.1186/1752-4458-4-28

**Published:** 2010-11-25

**Authors:** Robert Henley, Kate McAlpine, Mario Mueller, Stefan Vetter

**Affiliations:** 1 University of Zurich - Centre for Disaster and Military Psychiatry, Militaerstrasse 8, 8021 Zurich, Switzerland; 2 Roho Ltd, PO Box 16058, Arusha, Tanzania

## Abstract

**Background:**

This paper is based on data gathered from a 2006 survey of 1,098 "street children" in Northern Tanzania. It examines the role that school may play in preventing the migration of vulnerable youth to become homeless "street children". Specific focus is placed on the correlations found between children's attendance in school, their reports of abuse or support in their family, and their status of living "on the street" full-time or part-time.

**Methods:**

This study is from quantitative interview data gathered from 1,098 children and youth between 5 and 24 years old on the streets of Moshi and Arusha, Tanzania, over a 48-hour period during the school year on October 26^th ^and 27^th^, 2006. Respondents were given survey questions about their home, school and street life experiences, in order to measure the impact of outreach work being performed by a Tanzanian NGO. Interviewers used purposive sampling, approaching all young people who appeared to be under the age of 25 years within a number of precincts in each town known to be where 'street children' were known to congregate.

**Results:**

Results suggest that regular attendance in school may be a significant protective factor for children in preventing migration to the street life. Statistical analysis revealed that those young people who dropped out of school had nearly 8 times higher chances for ending up on the streets permanently than those who attended school daily.

**Conclusions:**

This study supports the new concept of "multi-layered social resilience", providing evidence from research completed by one NGO on how community-based organizations can help enhance resilience in a broader social context, spanning individuals, households and community structures.

## Background

### Street Children, School Attendance and Educational Resilience

It is estimated that as of 2009, over 50% of the world's population lives in urban areas, and this global urbanization process is having particularly profound effects on the physical and mental health of children who live there [1]. One of the more visible negative symptoms of urbanization is the growing numbers of children and youth who prematurely leave their families to live and work on the streets without homes [2,3]. Compounding the already difficult life circumstances of these young people is the fact that in many places homeless children are treated as societal outcasts, rather than being recognized as in need of care and protection. Indeed, the United Nations Convention on the Rights of the Child [4] recognises children as rights holders who should be supported to enact change in their own lives, no matter where they live or what their situations are.

As a response to this epidemic of youth homelessness internationally, organizations tend to focus on offering services to assist these "street children" with a goal of removing them from the streets and re-integrating them with their families and communities. There are many methods being utilized to contact and engage with street children on the streets, including offering sports, play, non-formal education and vocational training programs to get them involved and eventually off the streets. This paper will examine the work of one youth agency, Mkombozi http://www.mkombozi.org, a local NGO that has operated for over 12 years in the northern regions of Kilimanjaro and Arusha in Tanzania. Mkombozi has offered a variety of outreach services to prevent children coming to the streets, collaborating with local families, schools and community leaders to identify youth who may be vulnerable of becoming homeless and establishing community support mechanisms. Mkombozi's efforts illustrate how NGO's can contribute to building equitable development and long-term poverty eradication for citizens of all ages in low-income countries.

The Mkombozi organization hypothesizes that children's attendance in school plays a particularly crucial role in keeping them engaged and living in their communities, and thus preventing them from migrating away to live and work on the streets of urban areas. In Tanzania school attendance is compulsory but free for elementary school children, and free for all children that wish to attend. Unfortunately, there are additional costs for families to send their children to school, such as the purchase of uniforms and books. For poor families these additional costs can be prohibitive, which can inhibit their ability to afford sending their child to school.

Many youth are under pressure to leave school prematurely because of family poverty, whilst others are tempted to spend time on the streets during the day by the prospect of freedom and lack of supervision [5]. However, over time this "part-time" street lifestyle can eventually lead to a "full-time" street life, as a youth begins to disconnect from his/her family and focus on self-support (and reaping the full benefit of their work efforts). A "full-time street child" refers to children who live and work full time on the street, and who have very little or no regular contact with their family; "part-time" street children are those young people who still live with their families, but work and pursue other activities on the street during much of each day and night [6-8]. Lacking formal education, vocational training or other opportunities for regular employment, a child or youth who leaves home prematurely is a high risk for a life of low-income work and long-term poverty. Involvement in education and/or vocational training is therefore posited as a crucial protective factor, not only for the young person, but also for the community and society they could make contributions to.

The Mkombozi organization has identified the need to understand more precisely the forces pushing children to the streets and to test its assumption that keeping children in school would keep them off the streets. Thus, in 2003, 2005 and 2006 Mkombozi implemented a series of census-surveys in order to begin to (1) measure the impact of their preventive work; (2) to estimate the numbers and trends in the "street children" population; and (3) to examine the survival strategies that the street children use [8].

This paper is based on the statistical analysis of a second subset of data gathered during the school year from 1,098 children and youth from the 2006 census-survey of "street children" in the urban areas of Arusha and Moshi, Tanzania. The first subset of data identified where children lived - at home, part-time or full-time on the streets - and whether they reported having supportive or abusive relationship with their families [9]. This current analysis pays more specific attention to survey sub-section questions that (1) examined a child's current or past school attendance, (2) identified their reports of abuse or support in their family, and (3) identified whether they were currently living "full-time" or "part-time" on the streets.

### Individual psychosocial resilience

Conceptually, this paper examines the influence that school attendance has on children and youth from the theoretical framework of individual "resilience". The construct of individual psychosocial resilience has evolved from extensive research in the developmental psychology field [10-14], and is defined as: *"a dynamic process encompassing positive adaptation within the context of significant adversity. Implicit within this notion are two critical conditions: 1.) Exposure to significant threat or severe adversity; and 2.) the achievement of positive adaptation despite major assaults on the developmental process" *[15]. In this context, resilience capacities enables survivors of high-risk environments to develop, maintain or enhance social competence, empathy, caring, problem-solving skills, critical and creative thinking, task mastery, and a sense of purpose and social connectedness in the face of severe adversity and distress [16]. In this "resilience in the face of adversity" context, resilience can also be understood from the perspective of a "profile of adaptation", or as a "trajectory of adaptation", meaning that resilient behavior results in outcomes that may be markedly better from what might be normally expected [15,17].

Much literature on street children tends to emphasize the physical and emotional risks that the lifestyle incurs, and to examine how they cope in the face of such adversity, but it does little to examine the extent of their positive adaptation in the face of that environment and what skills and behaviors may be nurtured by that environment [18-20].

Current resilience researchers are urging more attention be paid to how "resilience processes" can be enhanced in young people via their involvement in structured sport/play, educational and vocational training programs [21,22]. It is believed that the encouragement of problem-solving skills in children and youth can be a particularly strong predictor of improved resilience over the long-term, as improved problem-solving skills can enhance the possibility that future life challenges will be resolved successfully [16]. Thus, beyond the obvious educational benefits of learning that school attendance can provide, particular attention must also be paid to the key social protective-enabling factors in school settings (e.g., relationships with peers, teachers, coaches, and trainers in a community setting) that could potentially help young people overcome the adversities that they face. These relationships offer the opportunity to provide young people with encouragement, and to support the development of their mental and social capacities, thus helping them to discover and experience new healthy trajectories in their lives [17,22-25]. Schools also expose adolescents to opportunities that build their sense of efficacy through achievement in sports, arts and academics, whilst positive peer relationships support young people to nurture a positive self-identity and to contribute to wider social realms. Unfortunately, attendance in school does not always result in the development of positive relationships with teachers or peers, nor helps to develop problem solving skills or other adaptive strategies, so this is an area that schools need to focus more on.

Adolescence is a time of rapid cognitive, social, emotional and physical changes, with particularly dramatic changes in pathways of adaptation occurring during the late adolescent period of emerging adulthood [26]. In this developmental phase, adolescents often lack the ability to cope when they experience stress, and this is often expressed in family and school environments [27]. The potential of school to assist in social and emotional development, is because it is a community-based setting where adults and peers can help cultivate resilience amongst young people, where interventions can be especially instrumental in influencing young people as they move from childhood into adolescence, from school into the workforce, and from youth into adulthood [15,17,22]. A key component of resilience development involves "envisioning the future", which includes *"feeling competent" *and *"elevating expectations" *that can be facilitated within the context of relationships with reliable, caring, and competent adults [28]. Preventive interventions can also focus on reducing risk and promoting protective factors in the child via their social contexts (e.g., in their family, classrooms, schools, peer groups, neighbourhood, etc).

### How Development NGOs Promote Social Resilience

The significance of social factors for enhancing the development of resilience processes in young people leads us to consider another important and new conceptualization of resilience, which involves the current work of Obrist, et al [29], who defines resilience in a larger "layers of social resilience" contextual perspective. This "layers of social resilience" model requires the study of individual capacities in interaction with households, social groups, communities, public and private organizations, the outcome of which can strengthen both the individual and collective resilience through collaborative, interactive effects.

Obrist further defines social resilience as *"*the capacity of actors to not only cope with and adjust to adverse conditions (i.e. reactive capacity) - but search for and create options (i.e. proactive capacity), and thus develop increased competence (i.e. experience positive outcomes) in dealing with threats" [29]. Social resilience in this context thus involves planning, preventing, evading, mitigating, avoiding, as well as coping with and reacting to challenging livelihood conditions. It refers to proactive capacities like anticipating, changing and searching for new options. Therefore, in contexts of adversity, a learning process that promotes and results in healthy behavioural adaptation and adjustment is an essential dimension of resilience, and leads to increased competence in dealing with challenging livelihood conditions.

## Methods

### Sample

This study sample is from quantitative interview data gathered from a population of 1,098 children and youth between 5 and 24 years old in Northern Tanzania, with 70% of the respondents between the ages of 10 and 19 years old, 78.5% of who are males. Respondents were given survey questions about their home, school and street life experiences, in order to measure the impact of outreach work being performed by the Tanzanian NGO "Mkombozi". Data was collected through on-the-street interviews with all children and young people visible on the streets in the towns of Arusha and Moshi in northern Tanzania, during the school year over a 24-hour period on October 26^th ^and October 27^th^, 2006. Interviewers used purposive sampling, approaching all young people who appeared to be under the age of 25 years, within a number of precincts in each town known to be where 'street children' were known to congregate.

### Ethics Review

The census-survey was conducted with the approval of the local Moshi and Arusha Government Social Welfare departments in northern Tanzania, who believed that the information gained by interviewing homeless children was a significant public health benefit. The study was then given a retroactive ethics clearance for publication by the Tanzania National Institute of Medical Research (NIMR). Finally, once the ethics clearance was approved by NIMR, it was as well approved by the State (canton) of Zurich ethics review process.

Each child or youth approached by interviewers were provided information about the nature of the study, and asked for their "informed consent": where all interviewed young persons were first informed of their rights to participate in an interview or not, to be able to refuse to answer a question or be able to end the interview at any time, and that in all circumstances their wishes would be respected and questioning stopped. If informed consent was not received from the child, they were not interviewed. Any interviewees were also given information about whom they could talk with for counselling support, should they desire further support anytime after the interview. All data was collected anonymously, with no details of names or other identifying information asked for or retained. Further, the data sets shared with the University of Zurich were sent as completely anonymous secondary data on electronic excel spreadsheets.

### Interviewing procedure

Collection of field research data with street children is complicated by the fact of these children being "restless and reluctant responders" who are generally mistrustful of unfamiliar adults [30]. To encourage participation in this survey, Mkombozi used former street youth over the age of 18 to deliver a questionnaire to persons they identified as under the age of 25 years who were visible on the streets, with the support and supervision of Mkombozi staff supervisors. The interviewers participated in trainings prior to the survey dates, as well as in post-survey debriefing sessions. Since literacy rates are low among these children, the interviewers read questions, presented the response options and then recorded the children's responses.

### Instrument

A questionnaire was developed in English, then translated into Swahili and then back-translated into English with further refinements among bi-lingual members of the Mkombozi staff. The questionnaire was then pilot-tested by giving it to former street children who were in care at a residential centre to assess face validity. The questions disaggregated children living on the streets full time from those who had only partial contact with the following questions: "Do you live and sleep on the streets all of the time, both day and night?" In response to a negative reply they were asked: "Do you come to the streets during the day and return home at night?" Interviewees were also asked what activities they engaged in, and to rate the range of time spent on the street using a four point Likert-type scale. There was also a section in the survey that asked questions about school attendance. For example, "Are you a primary school student?" (yes or no), and if yes, "Are you attending daily", "Playing truant", or "Have dropped out".

Lastly, possible supportive and abusive factors in family homes were assessed by inserting eight Likert-type questions adapted from the Adverse Childhood Experiences (ACE) test [31], which were used to collect information on childhood maltreatment, household dysfunction, and other socio-behavioural factors examined in the Adverse Childhood Experiences Study. The ACE [31] was chosen because it was a template of what sort of questions could surface information about abuse or support factors.

### Data

The survey collected data on demographic information, school attendance patterns, and an assessment of how the interviewee saw family life. This covered eight variables of family life addressing three positive items (i.e., respondents were asked if they saw their family as a source of strength, support, and protection) and five negative items (i.e., respondents were asked whether an adult or caretaker ever hurt them emotionally, physically or sexually, or whether they had ever witnessed this happening to someone else). When completing the questionnaire the children could rate the frequency of these experiences from never (0) to very often (3), with each of the variables being analyzed as single items. The self-reported information on whether children were living on the street (permanently or temporarily) at the moment, served as an outcome in following analyses.

### Statistical analysis

Street children status was numerically assigned in 3 ways, namely whether they self-identified as living "part-time" or "full time" on the street, or if they did not identify as living on the street at all (e.g., (1) "Not living on the street"; (2) "Living part-time on the street"; or (3) "Living full-time on the street"). These responses were considered as a polytomous outcome variable, with these three categories as possible answers. Explanatory variables were selected based on theoretical considerations. As displayed in Table 1 contingency tables were analyzed using chi square statistics for each categorical variable regarding its association to street status. T-test statistics were used to compare groups of street status in continuous variable scores. Because assumptions of parallel regression were violated for most variables, no ordinal regression model for ordered outcomes was conducted. Instead, multinomial logistic regression models were used to determine multivariable effects of explanatory variables on those categorical outcome variables that were found significant at p ≤ .05 in univariate analyses. Therefore a hierarchic sequence of three consecutive models has been carried out (see Table 2). After controlling for sex and age (Model 1) additional influences of abusive and supportive factors in family life (Model 2), and lastly school attendance (Model 3) were estimated. Nominal variables were broken into two or more dummy variables, depending on the number of categories. Therefore, the influence of every single category could be estimated. The omitted category (= 0; as defined in Table 1) of these variables served as the reference category, and continuous variables were centered using z-standardized mean scores. That allows for estimating odds ratios for groups in varying variable scores around the sample mean score. P-values of 0.05 or less were considered statistically significant. Odds ratios were estimated in the logistic regression and 95% confidence intervals were calculated on these estimates. All data analyses were performed using Stata 10.1 for Macintosh [32].

**Table 1:** Proportions of demographic features, distributional aspects of support-/abuse-variables, and their bivariate associations with street-status

	TOTAL BASE SAMPLE	REFERENCE SUB-SAMPLE	TEST SUB-SAMPLES		TEST
		**Never been on the street**	**Part-time on the street**	**Full-time on the street**	**P-value**

*Male (a) N (%)*	862 (100.0)	482 (55.9)	279 (32.4)	101 (11.7)	< .001
	
*Female (a) N (%)*	236 (100.0)	180 (76.3)	47 (19.9)	9 (3.8)	

*Attending daily (a) N (%)*	663 (100.0)	502 (75.7)	136 (20.5)	25 (3.8)	< .001
	
*Playing truant (a) N (%)*	185 (100.0)	103 (55.7)	50 (27.0)	32 (17.3)	
	
*Dropout (a) N (%)*	250 (100.0)	57 (22.8)	140 (56.0)	53 (21.2)	

*Age 5-9 N (%)*	185 (100.0)	158 (85.4)	23 (12.4)	4 (2.2)	< .001
	
*Age 10-14 N (%)*	432 (100.0)	303 (70.1)	88 (20.4)	41 (9.5)	
	
*Age 15-19 N (%)*	339 (100.0)	156 (46.0)	136 (40.1)	47 (13.9)	
	
*Age 20-24 N (%)*	142 (100.00)	45 (31.7)	79 (55.6)	18 (12.7)	

***Total ****N (%)*	**1098 (100.0)**	**662 (60.3)**	**326 (29.7)**	**110 (10.0)**	

*Emotional abuse (b)*	.85 (1.14)	.74 (1.10)	.93 (1.16)	1.27 (1.25)	< .001

*Physical abuse (b)*	.89 (1.09)	.73 (1.03)	1.02 (1.12)	1.40 (1.15)	< .001

*Sexual abuse (b)*	.63 (1.00)	.53 (.95)	.74 (1.01)	.99 (1.14)	< .001

*Trauma (b)*	.78 (1.01)	.67 (.97)	.90 (1.04)	1.06 (1.09)	< .001

*Neglect (b)*	.82 (1.22)	.69 (1.13)	.94 (1.29)	1.37 (1.31)	< .001

					

*Family love (b)*	1.83 (1.18)	2.04 (1.10)	1.66 (1.18)	1.11 (1.26)	< .001

*Family protection (b)*	1.77 (1.18)	1.98 (1.11)	1.59 (1.17)	.97 (1.21)	< .001

*Enough food (b)*	1.75 (1.19)	1.97 (1.14)	1.52 (1.17)	1.12 (1.17)	< .001

^a^values are proportions, group comparisons are based on Chi square statistics

^b^values are mean scores, group comparisons are based on analyses of variance (ANOVA)

^c^sub-proportion compared to those who have never been on the streets

**Table 2:** Results of hierarchic multinomial logistic regressions: sequences of risk factors for street-status

	LIVING TEMPORARILY ON THE STREETS (C)			LIVING PERMANENTLY ON THE STREETS (C)		
	**OR (95%CI)**			**OR (95%CI)**		

	**Model 1**	**Model 2**	**Model 3**	**Model 1**	**Model 2**	**Model 3**

*Female (a)*	**.43 (.30-.64)**	**.44 (.30-.65)**	**.42 (.27-.63)**	**.25 (.12-.51)**	**.22 (.10-.48)**	**.21 (.09-.47)**

						

*Age 10-14 N(%) (a)*	**1.80 (1.09-2.97)**	**1.77 (1.06-2.96)**	1.42 (.84-2.41)	**4.60 (1.61-13.14)**	**3.90 (1.33-11.43)**	**3.16 (1.05-9.51)**

*Age 15-19 N(%) (a)*	**5.51 (3.35-9.07)**	**5.20 (3.12-8.66)**	**3.40 (2.00-5.78)**	**10.57 (3.70-30.16)**	**8.10 (2.75-23.84)**	**5.36 (1.77-16.26)**

*Age 20-24 N(%) (a)*	**11.96 (6.72-21.30)**	**10.41 (5.76-18.82)**	**6.75 (3.65-12.49)**	**15.62 (4.99-48.82)**	**10.29 (3.17-33.38**	**7.05 (2.11-23.63)**

						

*Emotional abuse (b)*	-	.88 (.72-1.07)	.84 (.68-1.04)	-	.86 (.64-1.15)	.82 (.61-1.10)

*Physical abuse (b)*	-	**1.21 (1.00-1.47)**	1.19 (.97-1.45)	-	**1.41 (1.06-1.87)**	**1.36 (1.02-1.82)**

*Sexual abuse (b)*	-	1.05 (.88-1.26)	1.03 (.86-1.25)	-	1.10 (.85-1.42)	1.09 (.84-1.42)

*Trauma (b)*	-	1.12 (.95-1.33)	1.11 (.92-1.32)	-	1.12 (.87-1.44)	1.07 (.83-1.40)

*Neglect (b)*	-	1.04 (.89-1.23)	1.05 (.89-1.24)	-	**1.34 (1.07-1.68)**	**1.30 (1.03-1.64)**

						

*Family love (b)*	-	**.80 (.66-.98)**	.87 (.71-1.08)	-	**.70 (.52-.94)**	.77 (.57-1.04)

*Family protection (b)*	-	1.05 (.85-1.30)	1.12 (.90-1.40)	-	**.71 (.51-.98)**	.77 (.55-1.08)

*Enough food (b)*	-	**.82 (.68-.98)**	**.83 (.69-1.00)**	-	**.74 (.57-.98)**	.78 (.59-1.04)

						

*Playing truant (a)*	-	-	1.27 (.84-1.94)	-	-	**3.21 (1.75-5.90)**

*Dropout (a)*	-	-	**5.47 (3.66-8.16)**	-	-	**7.85 (4.31-14.28)**

OR: Odds ratios, 95% CI: 95% confidence interval

Numbers in bold print are significant at p ≤ .05

^a^Estimated parameters are Odds Ratios towards the omitted factor level (reference category) of the concerning variable

^b^Odds Ratio estimate is for every increase of 1 unit in the variable score above the group mean score (see Table 1)

^c^Samples are sub-samples of those in the first column

## Results

As displayed in Table 1 the prevalence of total street children, as well as sub-samples by sex, age and school status were determined. These demographic features turned out to be significant indicators of street status in univariate analyses. Overall, males were more frequently living on the street than females. In detail, rates for males living full-time on streets are more than three times as high compared to females, while proportions for "part time" street children reflected the overall rates. Furthermore, "full time" street children more frequently reported being "truant" or "dropped out" of school than the "part time" street children did. Without exception, items of abusive and supportive factors are associated to street status in a clear trend. Accordingly, "part-time" and "full-time" street children had significantly higher abusive scores than those who have never been on streets, with an increasing tendency - and vice versa for supportive factors - children who have never been on streets had higher support scores than those children living on streets temporarily and permanently.

A hierarchic sequence of three multinomial logistic regression models was performed to determine risk factors for street life among children surveyed by Mkombozi. Street status served as outcome variable with "never been on streets" as reference category. Odds ratios were estimated for all variables that were significantly associated to street status in univariate analyses (see Table 1). All variables that were found to be independently associated with street status in the common model are displayed in bold in Table 2.

Gender and age significantly influence street status as an outcome. Females have a much lower chance of becoming a street child than males, especially in the chance of becoming a full time street child, with these odds for gender remaining stable in all three models. Furthermore, increasing age (in categories) is significantly associated with increased "living on the street" status in all models (i.e., more older youth live on the street). Also, "full-time" street life is more highly associated with age than "part-time" street life (i.e., older youth are more likely to be "full-time").

When adding self-reported supportive and abusive factors to the equation (model 2), lower level associations were found for reports of physical abuse and neglect as additional risk factors for street life, while the latter was only associated to permanent street life. However, reports of higher "family love", "family protection", as well as "having enough food at home", turned out to be important protective factors against ending up on streets, both temporarily and permanently, except for "family protection" which revealed no elevated risk for part-time street life.

The final model (model 3) revealed that school attendance plays a crucial prevention role in the likelihood of a child ending up living on the streets, independently of all other factors. A child dropping out of school, as well as being truant, is highly associated with taking up a life on the street, and especially a "full-time" street life. Whereas, being truant had no associations to part-time street life. Those who dropped out of school had nearly 8 times higher chances for ending up on the streets permanently than those who attend school daily, while the odds for migrating to the streets temporarily is only a bit lower (OR = 5.5). In this last model, most former associations between support/abuse and street status disappeared. Having enough food was sufficiently protective against a part-time but not full-time street life. Reports of physical abuse and neglect (whether at home or at school) remained significant risk factors for migration to permanent street life.

## Discussion

The key findings from the statistical analysis of the survey results gathered from 1098 children and youth on the streets in the urban areas of Moshi and Arusha in Tanzania, supports the hypothesis that school attendance plays a crucial protective role in preventing children from migrating to live on the streets. In Tanzania attendance in school is compulsory but free for elementary school children, and free for all children that wish to attend beyond that, although there are additional costs (e.g., books and uniforms) that can inhibit a poor families' ability to pay for a child to attend school.

The results suggest that children who drop out of school are highly associated with becoming homeless, and highly correlated with taking up a "full time" street life. Those who dropped out of school have an eight times higher chance for ending up on the streets, compared to those who attend school daily. Therefore, beyond the obvious benefits of gaining an education (in the form of knowledge and competencies), significant positive developmental outcomes are also gained from participation in a school environment, having to do with the mental, social and behavioural health benefits that engagement in a school community can provide. School attendance was not only found to be a significant protective factor in preventing migration to street life, it was also found to be a significant protective factor in counteracting the negative factors of abuse, trauma and neglect that some children experience in their homes.

There are some significant limitations to this research: The subjects were not randomly selected, there wasn't a comparison group, and the survey did not gather enough precise information to provide a more detailed understanding of whether the sources and effects of support or abuse in the home, in the school, and/or on the streets. We don't know if the children went to the street and then dropped out from school, or vice versa, and to what extent violence in school caused the migration to the street (e.g., corporal punishment is still used by some teachers in Tanzania). More information could have been gathered about the role that school plays from the perspectives of the "street children". And lastly, the data that was collected was not originally intended to be used for published empirical research, but rather, as a means to monitor and evaluate the effectiveness of an NGO's implemented program services.

Nonetheless, this census-survey undertaken by the NGO Mkombozi is important for the new knowledge that was gained and shared with their local communities. For example, Mkombozi has been able to adapt its services and interventions based on the survey data gathered, allowing them to identify specific neighbourhoods and schools that had more school attendance problems, thus enabling them to identify and focus on implementing the most effective prevention practices for support of individuals, families, schools and communities. Anecdotal information was also provided by Mkombozi about how the collected data was shared with school and community leaders, who either intervened with families who made their children go to work rather than allow them to attend school; or who developed creative ways to support those in the community who couldn't afford the extra fees required to enrol their children in school. In one example, a school started selling lunches on-site, and with the profits paid a local tailor to sew new school uniforms for the children of families who couldn't afford to buy these for their children, thus enabling the children to attend school.

The results of this research, despite the above noted limitations, offer glimpses into the key role that educational can play in enhancing individual, organizational and community resilience to adversity, and in turn improve opportunities for more successful and sustainable development. It is strongly recommended that more research be focused on how school attendance and participation can enhance the psychosocial strengths and competencies of children and youth to more successfully meet the challenges they will face.

## Conclusion

The significant statistical correlations found between "full time" street status, reports of supportive or abusive households, and school attendance provides support for the "multi-layered social resilience" conceptual model. The practices of the NGO Mkombozi, originally founded to help individual street children "on the street", have been adapted and expanded to offer improved educational services for troubled children in a number of contexts: 1). For those still living with their families and attending school regularly. 2. To provide informed outreach and advocacy services to more effectively engage, collaborate with, and encourage increased community involvement with its families and children. 3. And to both educate and support the efforts of local health and law enforcement agencies in more effectively addressing the root problems faced by "street children" in their community context. This is "multi-layered resilience" in action: a community organization working to improve the quality of life for individuals, households, and community structures. These research efforts have resulted in helping Tanzanian communities to be more resilient through strengthening collective coping skills (i.e., "reactive resilience"), and also through the development and strengthening of local problem-solving strategies (i.e., "proactive resilience"), thus enhancing resilience competencies on many layers: for the individual, household, community, regionally, and potentially, even nationally.

## Competing interests

The authors declare that they have no competing interests.

## Authors' contributions

RH: Conceptual development, principal writer, resource research, data analysis, data interpretation, and editing. KM: Conceptual development, designer, principal investigator and writer. MM: Investigator, statistical analysis and interpretation. SV: Investigator, writer and final editing. All authors read and approved the final manuscript.
